# Public Health Policy Responses to Population Aging in Mexico: A Rights-Based and Socio-Legal Approach

**DOI:** 10.3390/healthcare14182944

**Published:** 2026-09-10

**Authors:** Patricia Rojas, Carolina Rojas, Aída Rojas-Castañeda, Margarita Martínez Gómez, María Esther Ocharan-Hernández, Judith Pacheco-Yépez

**Affiliations:** 1 Centro Tlaxcala de Biología de la Conducta, Universidad Autónoma de Tlaxcala, Av., Universidad s/n, Col. La Loma Xicohténcatl, Tlaxcala C.P. 90070, Mexico; marmag@biomedicas.unam.mx; 2 Departamento de Biología Celular y Fisiología, Instituto de Investigaciones Biomédicas, Universidad Nacional Autónoma de México, Circuito Escolar s/n, Ciudad de México C.P. 04510, Mexico; rocastan@yahoo.com.mx; 3 Facultad de Derecho, Universidad Nacional Autónoma de México, Circuito Interior s/n, Ciudad de México C.P. 04510, Mexico; arccourses24@outlook.com; 4 Departamento de Biología Celular y Fisiología, Tlaxcala Unit, Instituto de Investigaciones Biomédicas, Universidad Nacional Autónoma de México, Av., Universidad s/n, Col. La Loma Xicohténcatl, Tlaxcala C.P. 90070, Mexico; 5 Sección de Estudios de Posgrado e Investigación, Escuela Superior de Medicina, Instituto Politécnico Nacional, Plan de San Luis y Díaz Mirón s/n, Col. Casco de Santo Tomas, Ciudad de México C.P. 11340, Mexico; estherocharan@gmail.com (M.E.O.-H.); jpachecoy@ipn.mx (J.P.-Y.)

**Keywords:** healthy aging, health promotion, health policies, social policy in Mexico, older persons, older adults, legal framework in Mexico, Inter-American Convention on the Human Rights of Older Persons

## Abstract

**Background/Objectives:** Population aging represents a major social and public health challenge in Latin America. Mexico has made significant advances in regulatory and policy frameworks. Notable progress includes accession to the Inter-American Convention on Protecting the Human Rights of Older Persons (IACPHROP). It also includes the enactment of the Law on the Rights of Older Persons, and institutional reforms for social and cultural inclusion. This review evaluates Mexico’s alignment of aging policies with IACPHROP standards using an integrated framework of social determinants, policies, and legal obligations. This approach has not been previously reported for Mexico. **Methods:** Searches were conducted in electronic databases and official repositories. These included the World Health Organization, the Organization of American States, the Inter-American Human Rights System, and Mexican government regulations. Additional searches were performed in PubMed, Redalyc, SciELO, and Google Scholar. **Results:** Advances include universal non-contributory pensions, the expansion of healthcare coverage, and social inclusion programs. Important policy areas remain to be addressed, including the establishment of a national long-term care system, and the reduction of regional disparities in health coverage. In addition, the evidence linking specific policies to improve health outcomes remains heterogeneous. **Conclusions:** Mexico has built a solid foundation for the protection of older adults’ rights. The development of a long-term care system and the adoption of place-based approaches are also essential. Regional cooperation and community traditions play a pivotal role in achieving dignified, inclusive, and sustainable aging.

## 1. Introduction

The increase in life expectancy is considered one of the most significant demographic phenomena of the twenty-first century. Globally, the population of older adults has been increasing rapidly due to advances in public health, declining mortality rates, and various socio-environmental changes [1]. According to the World Health Organization (WHO), an older adult is defined as any individual aged 60 years or older [2].

In this context, life expectancy at birth has risen significantly. It increased from 46.4 years in 1950 to projections of 73.5 years by 2025, 77 years by 2050, and 81.7 by 2100 [3]. This demographic change has been observed primarily in high-income countries but has been also extended to middle- and low-income regions, including Latin America and the Caribbean. Within this region, countries such as Chile, Uruguay, and Cuba reported the highest life expectancy [3,4], reflecting notable progress. Nevertheless, structural inequalities persist, limiting the quality of life in old age. This underscores the importance of linking longevity with quality of life, psychological well-being, and equitable access to health services.

In Latin America, the process of demographic aging is unfolding within a context marked by significant economic, health, and territorial inequalities. Several factors have contributed to the reconfiguration of the region’s demographic structure, including the sustained decline in the total fertility rate. The data indicate a decline from 4.85 live births per woman in 1950 to 2.25 in recent years [5,6]. Additional factors contributing to this phenomenon include accelerated urbanization and international migration [7,8,9]. These dynamics have reshaped the structures and functions of families, communities, and institutions. At the same time, demographic aging is exerting a direct influence on the autonomy and living conditions of older adults, underscoring the urgent need for comprehensive public policies that are designed to protect their well-being and rights.

For this purpose, the Inter-American Human Rights System (IAHRS) has been recognized as a fundamental regional reference for the development of legal standards aimed at protecting this population group. The Inter-American Convention on Protecting the Human Rights of Older Persons (IACPHROP) [10], together with various resolutions of the Inter-American Commission and pronouncements of the Inter-American Court, provides essential instruments to guide States’ action and to evaluate compliance with its obligations in this domain.

On that basis, this review is necessary because Mexico acceded to the IACPHROP in 2023. Therefore, the government’s new obligations must be understood from a public health perspective. Our aim is to identify whether Mexico’s legal and policy instruments comply with IACPHROP standards for healthy aging. We also seek to determine areas of convergence, legal gaps, and opportunities to strengthen institutional commitment in promoting healthy, safe, and active aging.

Our analysis addresses three dimensions: the social determinants of health, social policies, and Mexico’s legal obligations for aging. This comprehensive approach has not been previously studied for the Mexican case, as some of these elements have been reported separately in the literature. Furthermore, Mexico’s experience could serve as a reference for other Latin American countries facing similar demographic and legal transitions.

## 2. Methods

### 2.1. Design

This study was conducted as a narrative review, employing a structured search strategy followed by a thematic synthesis. This methodological approach was selected for its suitability for integrating evidence from diverse and heterogeneous sources. This included peer-reviewed literature, legal and policy instruments, institutional reports, and official statistical data. The convergence of these materials provides a comprehensive analysis of public health policies and legal frameworks on aging in Mexico. It also serves to situate national developments within the broader Latin American context.

### 2.2. Search Strategy

We searched academic databases, including PubMed, Redalyc, SciELO, and Google Scholar. In addition, institutional repositories from the WHO, the Organization of American States (OAS), the Economic Commission for Latin America and the Caribbean (ECLAC), the World Bank, and the International Labour Organization were consulted. Mexican legal instruments and public policy documents were obtained from official Mexican government websites.

The search strategy used Boolean operators (“AND” and “OR”) in combination with key terms comprising “older adults”, “aging population”, “human rights”, “social rights”, “Inter-American human rights system”, “Mexico legal framework”, and “public policies on aging”. The search covered documents published between January 2015 and June 2026. This period was selected for four reasons: (i) the adoption of the Inter-American Convention on Protecting the Human Rights of Older Persons in 2015; (ii) it encompasses the evolution of major Mexican social programs; (iii) demographic data from the 2020 Mexican Census are available; and (iv) recent national surveys are available, particularly the Mexican Health and Aging Study (ENASEM) and the National Survey of Demographic Dynamics (ENADID). Both English and Spanish publications were considered for inclusion.

### 2.3. Eligibility Criteria

Inclusion criteria comprised peer-reviewed articles, official legal texts, institutional reports from recognized organizations, and official statistical data, if they addressed Mexico, Latin America, or the Caribbean. Global documents were considered when they provided comparative frameworks relevant to the region. Furthermore, we prioritized sources that integrated health promotion, equity, and biopsychosocial perspectives.

Exclusion criteria included opinion pieces, conference abstracts, non-peer-reviewed materials, and studies focused exclusively on countries outside the Americas without comparative relevance. Additionally, sources with a primarily clinical orientation and unrelated to public policy or human rights frameworks were excluded.

### 2.4. Screening and Selection

The selection process followed a multi-stage procedure to minimize bias. The initial searches of databases and repositories identified 395 records. After removing duplicates (55 records), 340 remained for selection. Two independent reviewers evaluated titles and abstracts using the predefined eligibility criteria. In cases of disagreement between reviewers, the issue was resolved through discussion, with a third reviewer consulted when necessary. From the 340 screened records, 145 were excluded for being outside the scope of the review or were not directly relevant to the research question. The remaining 195 records advanced to full-text evaluation, with the same review procedure and exclusion reasons documented. At this stage, 65 were excluded because they had a clinical focus irrelevant to policy, were from outside Latin America without comparative value, or were opinion pieces, or conference abstracts. Finally, 130 sources met the inclusion criteria and formed the basis of the thematic synthesis. To ensure completeness, the reference lists of included articles were manually searched for additional relevant sources.

### 2.5. Quality and Credibility Assessment

For quality and credibility appraisal, a source-based approach was adopted as follows: (i) peer-reviewed literature was prioritized when published in indexed journals with established academic reputations; (ii) official documents were evaluated according to the authority of the issuing body; and (iii) demographic and statistical data were exclusively obtained from official institutions, such as the National Institute of Statistics and Geography (INEGI) and the United Nations Population Division. No formal risk-of-bias assessment was conducted, as such instruments are not standard practice for narrative reviews. However, sources were not considered as equivalent and were classified according to their evidentiary role (e.g., reviews, original articles, legal documents, and institutional reports). Thematic triangulation across these source types was applied to strengthen the validity of our findings.

### 2.6. Thematic Synthesis

The selected literature was organized thematically using a combined deductive-inductive approach. Deductive categories were derived from established concepts in public health policy, human rights, and social determinants of health. Inductive categories emerged from the literature and encompassed Mexican policy developments, regional cooperation, and cultural dimensions of aging. These thematic categories structure the results section and highlight the evidence connecting rights-based frameworks with functional ability, autonomy, and social participation in later life.

## 3. Results

### 3.1. Population Aging in Latin America and the Caribbean

Latin America and the Caribbean are undergoing significant demographic transformation. While overall population growth has slowed, the proportion of older adults has continued to rise steadily. This process is reflected in a structural reconfiguration of the population pyramid, with far-reaching economic, social, and political implications. Recent studies indicate that the population aged 60 and above increased from 8.7 million in 1950 to 89.9 million in 2023. This represents 11.7% of the region’s total population. But demographic projections indicate that this trend will persist, with an estimated 188.1 million older adults in 2050, equivalent to approximately 25.1% of the regional population. At the same time, the population under the age of 15 is projected to decline to 17.4% in the coming decades [11,12]. If these trends continue, within two decades older people will outnumber children aged 0–14 years, marking a historic turning point in the demographic structure of the region.

This phenomenon is explained primarily by the sustained increase in life expectancy, which in the Latin American and Caribbean region increased from 46 years in 1950 to 74.3 in 2023, with projections estimating 79.3 years by 2050 [11,12]. These advances are attributed to improvements in health systems, nutrition, and access to basic services. However, they also highlight persistent inequality gaps between countries and within each nation [11,12]. In countries such as Chile, Uruguay, and Cuba, life expectancy exceeds 77 years. In contrast, other nations in the region continue to confront significant issues related to health, social security, and quality of life. This comparison demonstrates that the challenge is not only quantitative but also qualitative, requiring that increased longevity be accompanied by dignified living conditions and equitable access to services.

Older adults’ health and well-being indicators were adversely affected by the COVID-19 pandemic, which further exacerbated vulnerabilities [13,14]. Furthermore, recent studies conducted in Mexico and other regions have reported the adverse effects of prolonged isolation on mental health and quality of life [14,15].

In this context, the process of aging in Latin America and the Caribbean is recognized as a multidimensional phenomenon which is influenced by demographic, economic, and cultural factors [16,17]. It has been shown that older people are exposed to adverse social determinants, such as job insecurity, inadequate income, age discrimination, and limited coverage of social protection systems. This scenario emphasizes the need for comprehensive public policies that are designed to ensure autonomy, social inclusion, and improved quality of life in old age, in accordance with the principles of active aging promoted by the WHO.

In contrast to developed economies, where population aging has occurred after sustained economic growth and the establishment of extensive social safety nets, Latin America is undergoing this process without comparable levels of economic and institutional development. The region’s demographic transition is taking place in the context of structural inequality, labor informality, and weak social security systems. Consequently, the working-age population may contract before sustainable welfare mechanisms are consolidated [11,12]. This situation implies risks associated with loss of productivity and increased fiscal pressures on health and pension systems [18]. Another critical concern is the potential widening of gender gaps, as older women are often overrepresented in poverty and unpaid work [19].

Finally, the social determinants of health are linked to the living conditions of older adults, as they can either increase vulnerability or foster opportunities for healthy aging. These determinants are indicative of the interplay of structural factors, including wealth, the organization of the healthcare system, educational attainment, and the physical environment, among others, that determine well-being in later life. Moreover, the inequalities that have been accumulated over the course of life tend to be exacerbated in old age, which limits social participation and affects capabilities [20].

### 3.2. Social Determinants of Health Equity

Social determinants of health are the conditions in which people are born, grow, live, work, and age [21]. Health equity is contingent upon addressing the structural determinants that generate unequal health outcomes across population groups. These determinants include structural discrimination, which must be addressed with equal urgency. Likewise, the impacts of conflicts, emergencies, and migration warrant careful consideration. Discriminatory practices within public policies should be confronted through legal recognition and action. Such measures aim to reduce inequalities affecting women, girls, and indigenous communities [22,23,24,25]. Safe and inclusive environments are essential for reducing vulnerability. These environments also foster active participation among older populations.

Therefore, cross-sectoral cooperation is needed to improve international support for health equity. Accountability and respect for human rights are fundamental to this effort. The empowerment of local communities is also essential for sustainable change. These communities possess contextual knowledge and can lead effective actions [26,27,28,29,30,31,32]. A health system consolidation based on equity, citizen participation, and primary care can generate significant progress. However, this requires training health personnel in social and cultural issues and developing reliable information systems to monitor inequalities. Ultimately, health equity and healthy aging depend on social, economic, and cultural conditions. These factors shape well-being and functional ability in later life.

#### 3.2.1. Social Determinants Shaping Healthy Aging and Well-Being

Social determinants exert a direct influence on the quality of life, autonomy, access to services, and longevity of older adults. Economic stability, for example, is recognized as a critical factor, as low income has been shown to increase the risk of disability and premature mortality [33,34,35]. In this regard, several countries have implemented economic service programs to reduce vulnerability in old age.

Non-contributory pensions in Latin America and the Caribbean have gained relevance as a social determinant of health. This is due to two structural trends: rapid population aging and persistent labor market informality, which exclude millions from traditional contributory systems. From a public health perspective, these pensions are a key social determinant. By guaranteeing a minimum income, they enhance nutrition, access to medical services, and overall quality of life while simultaneously reducing vulnerabilities and gender inequalities [36].

Non-contributory pensions directed at older adults are economic benefits financed by the State, granted without the requirement of prior contributions to the social security system. Their purpose is to secure a basic income, prevent poverty in old age, and mitigate social inequalities. Two main modalities can be distinguished (Table 1):

(1) Universal, aimed at all older adults who meet basic age and residency requirements, regardless of their economic situation.

(2) Targeted, directed at specific groups defined by socioeconomic criteria (poverty, low income, absence of another pension) or categorical criteria (rural populations, Indigenous groups, or residents of specific areas).

Regional trends reveal a shift toward more inclusive models. Countries such as Mexico, Bolivia, and Guyana have implemented universal schemes covering more than 75% of the older population. In Chile, the Universal Guaranteed Pension approaches universality, although it excludes higher-income groups. In contrast, other countries maintain targeted programs with narrower reach and lower benefit levels, limiting their capacity to ensure dignified aging [36].

Mexico, the region’s second-largest economy, is paradigmatic. Its Pension for the Well-Being of Older Adults provides a basic income to individuals aged 65 and over, regardless of employment history. With coverage exceeding 75%, it has become a regional benchmark [36]. Its public health impact is significant, as it reduces economic vulnerability, improves access to health services, and helps close gender gaps, particularly benefiting women. This measure is regarded as representing meaningful progress in advancing social rights and promoting well-being. Nevertheless, it confronts obstacles concerning coverage, equitable distribution, and the cultural adequacy of services. Notably, the largest economies in the region, except for Mexico, have developed non-contributory pension models with more limited coverage. In Brazil and Argentina, programs are targeted and conditioned on the absence of contributory pensions, which restricts their effective reach. In Colombia, the fourth largest economy, the non-contributory pension scheme is also targeted, but eligibility does not require the absence of contributory benefits, thereby partially expanding coverage [36].

Taken together, non-contributory pensions in Latin America and the Caribbean represent a strategic instrument to confront the challenges posed by demographic aging and widespread labor market informality. Their reinforcement is essential to ensure economic security, reduce structural inequalities, and promote healthy and dignified aging. In this context, the role of the largest economies is particularly critical, as their social policy frameworks often set the regional agenda and provide benchmarks for the design of more inclusive and sustainable strategies.

It must also be noted that the social and community context plays a decisive role in the well-being of older adults, as loneliness and social isolation are associated with an increased risk of dementia and other serious diseases, while positive social relationships have been shown to contribute to better health outcomes and greater longevity [37,38,39,40].

Another important factor is that formal education and health literacy are essential for making informed decisions [41]. However, many older people encounter difficulties in interpreting medical documents, a problem that can negatively affect the quality of care they receive. Likewise, access to health services is limited by geographical barriers, high costs, and limited availability, particularly in rural areas. In addition, the physical environment is critical at this stage of life, as reduced mobility requires accessible and safe neighborhoods with nearby services that facilitate food shopping and physical activity [42]. These conditions show the importance of safe environments and equitable access to services as central pillars of healthy aging. These challenges are compounded in the context of international migration. Therefore, older adults face additional inequalities related to housing, economic stability, educational attainment, health literacy, social integration, and physical environment. In such circumstances, social determinants intensify health disparities and limit access to comprehensive and equitable care [43].

In Mexico, the number of older migrants settling has increased, whether due to personal preference, family reunification, or humanitarian need. This nation is no longer considered a transit place. It has become a destination for migrants, including U.S. retirees attracted by the climate, cost of living, and cultural proximity. At the same time, an additional group arrives through forced migration, mainly from Central America, the Caribbean, and South America. This population faces significant obstacles, including limited access to health services, language barriers, and difficulties with health literacy, social isolation, and the absence of community networks [44,45]. These barriers illustrate how social determinants intensify health disparities and limit equitable access to care for vulnerable older migrant populations.

#### 3.2.2. Comparative Indicators Among Older Adults in Six Latin American Countries

Mexico’s progress and difficulties in protecting the rights of older adults are presented in Table 2. This table provides a comparative analysis of key indicators for the population aged 65 and over in six Latin American countries. These are Mexico, Chile, Uruguay, Costa Rica, Brazil, and Colombia. These data provide quantitative context for assessing Mexico’s position within the region. It is to identify structural challenges that may hinder the translation of legal commitments into tangible improvements in quality of life.

Chile, Uruguay, and Costa Rica have the highest life expectancies at birth (78–81 years). They also have the largest shares of older adults (65 years and over) at 15%, 16%, and 13%, respectively. In contrast, Mexico has a life expectancy of 75 years and an older adult population of 9% (65 years and over), indicating a less advanced aging transition. This demographic lag may provide Mexico with additional time to prepare for population aging. However, it also underscores the urgency of implementing proactive policies. Projections indicate that by 2050, older adults will represent 22.5% of the population [46,47]. Mexico must act now before the aging process accelerates.

In terms of economic security, Chile leads in contributory pension coverage (87.3%), followed by Brazil (81.3%). Mexico (32.2%) and Colombia (27.7%) show the lowest rates. Nonetheless, Mexico’s non-contributory pension coverage exceeds 75%, representing a significant achievement in universal social protection. This level is comparable to Chile’s quasi-universal scheme. In contrast, Brazil, Colombia, and Costa Rica report coverage below 30%, often conditional on the absence of other pensions. This reveals different approaches to address old-age income security. Mexico shows low contributory coverage but high non-contributory coverage. This suggests a dual system where the State compensates for persistent gaps in labor market capacity. Nevertheless, this model may face fiscal sustainability challenges as the population ages.

On the other hand, Chile (11%) and Brazil (10%) have the highest health expenditures while Mexico (6%) has the lowest. In addition, Mexico has the lowest domestic general government health expenditure (3%), and Uruguay the largest (7%). Notably, out-of-pocket expenditure as a percentage of current health expenditure is highest in Mexico (41%) and Chile (39%). It is lowest in Colombia (15%) and Uruguay (17%). This high out-of-pocket burden in Mexico suggests that older adults face significant financial barriers to accessing healthcare. This could exacerbate health inequalities. It could also undermine the effectiveness of universal health coverage initiatives. But the Universal Health Coverage service coverage index is relatively consistent across all countries (79–85). This suggests that the provision of essential services remains similarly effective, regardless of spending levels. However, this may mask underlying inequalities in quality and access.

**Table 2:** Comparative indicators for older adults in selected Latin American countries ^1^.

Indicator	Mexico	Chile	Uruguay	Costa Rica	Brazil	Colombia
**Population**						
Life expectancy at birth (years) ++	75	81	78	81	76	78
Population ≥65 years (% total population) +++	9	15	16	13	11	10
**Economic security**						
Contributory pension for those aged ≥65 years (%)	32.20	87.30	34.90	50.60	81.30	27.70
Noncontributory pensions ≥65 years (%) *	>75	>75	<10	10–30	10–30	10–30
**Health system**						
Current health expenditure, public and private (% GDP)	6 (+)	11 (++)	9 (+)	7 (+)	10 (+)	8 (++)
Domestic general government health expenditure (% GDP) ++	3	5	7	5	4	6
Out-of-pocket % of CHE ++	41	39	17	24	26	15
UHC service coverage index ++	79	84	85	84	84	82
**Social determinants**						
Informal employment (total) ≥65 years (%) +++	79.64	57	70.60	75.60	59.80	83.80
Male informal employment ≥65 years (%) +++	79.17	53.90	70.30	73.90	59.40	84.16
Female informal employment ≥65 years (%) +++	80.64	63	71	80.46	60.47	83
Educational attainment (years of schooling), ages 65+ **	6.48	9.21	8.13	7.59	5.65	5.92
Male educational attainment (years of schooling), ages 65+ **	6.94	9.55	8.12	7.75	5.7	6.02
Female educational attainment (years of schooling), ages 65+ **	6.12	8.94	8.14	7.45	5.62	5.84
**Legal framework for rights protection**						
Ratification/accession of the IACPHROP	Yes	Yes	Yes	Yes	Yes	Yes
Legislation for older persons	Yes	Yes	Yes	Yes	Yes	Yes

^1^ Source: Prepared by authors based on data from ECLAC, IBRD, OECD, WHO, ILOSTAT, WCDGHC (references [36,48,49,50,51,52,53]). IBRD, International Bank for Reconstruction and Development; OECD, Organization for Economic Co-operation and Development; ECLAC, Economic Commission for Latin America and the Caribbean; WHO, World Health Organization; ILOSTAT, International Labour Organization Statistics; WCDGHC, Wittgenstein Centre for Demography and Global Human Capital; GDP, Gross Domestic Product; CHE, Current health expenditure; UHC, Universal Health Coverage; IACPHROP, The Inter-American Convention on Protecting the Human Rights of Older Persons; Educational attainment represents mean years of schooling; * Passive coverage, ** Data projections for 2025, + Data from 2023, ++ Data from 2024, +++ Data from 2025.

A critical structural challenge across the region is the high prevalence of informal employment among older adults. This is particularly pronounced in México (79%) and Colombia (83.80%) with lowest rates in Chile (57%) and Brazil (59.8%). Sex-based differences are minimal, with women showing slightly higher rates. Educational attainment for this population sector is highest in Chile (9.21 years) and Uruguay (8.13 years). It is lowest in Brazil (5.65 years) and Colombia (5.92 years). Mexico occupies an intermediate position (6.48 years). In all cases, women show slightly lower educational attainment than men.

The combination of informal employment and low educational attainment represents a significant barrier. It limits economic security, health literacy, and access to quality healthcare. This highlights the need for comprehensive social protection policies. Addressing educational disparities and labor market informality should be prioritized alongside pension reforms.

Finally, the six countries have ratified or acceded to the IACPHROP and have enacted specific legislation. These actions demonstrate a shared regional commitment to a rights-based approach, despite diverse sociodemographic realities. Moreover, legal ratification does not automatically translate into equitable outcomes. Countries with higher health expenditures appear to provide greater financial protection for older adults. For example, Uruguay has lower out-of-pocket costs than Mexico. This suggests that fiscal commitment to health and social protection is critical. It determines the effectiveness of policies.

This comparative analysis underscores that Mexico has made notable advances in universal pensions. But it faces challenges in public health financing, labor informality, and educational attainment. These areas must be addressed through an integrated policy approach. This should combine pension reforms, health system strengthening, and labor market formalization. By advancing these actions, Mexico can translate its legal commitments under the IACPHROP into tangible outcomes for its aging population.

### 3.3. Regional Human Rights Framework for Healthy and Active Aging

Social determinants have been shown to exert a direct impact on the well-being of older adults. In this regard, the increase in life expectancy in Latin America does not necessarily correspond to a parallel improvement in the quality of life of this population group. To address these challenges, collaboration among the OAS, the Inter-American Commission on Human Rights, and the ECLAC has been essential for consolidating a regional regulatory framework that responds to the social dimensions of aging and the protection of human rights. This cooperative effort culminated in the formulation of the Inter-American Convention on the Protection of Older Persons, a pioneering legal instrument that broadly and comprehensively recognizes the needs, vulnerabilities, and challenges faced by this population group.

The IACPHROP is recognized as a legally binding international treaty that specifically protects the rights of older people [10]. Within this framework, guidance is provided to States Parties in the design of public policies, programs, and legal structures that guarantee well-being, autonomy, and social inclusion. Moreover, the Convention establishes clear obligations for ratifying countries and promotes a rights-based approach, placing dignity, equality, and active participation of older people at the center of public and regional action, consistent with the WHO framework on healthy aging and the maintenance of functional ability. In this way, the Convention not only consolidates a robust regulatory framework but also represents an advance in the promotion of more inclusive and equitable societies for the older people in Latin America.

Among the countries that are part of the ECLAC, Mexico is noteworthy for its demographic significance, as it holds a position among the world’s most populous nations, ranking eleventh [3]. Additionally, the country’s economy is classified as the fourteenth largest globally. However, Mexico is undergoing a rapid aging process [54]. Thus, projections indicate that by 2050, the population aged 60 and over will represent approximately 22.5% of the total population, with a life expectancy of 80 years and a total fertility rate of 1.7 [3,46,47]. This demographic perspective underscores the strategic importance of fortifying the national system for the protection of the rights of older people in Mexico. Due to its population size and its status as an important emerging economy, institutional responses, particularly in the areas of public health, social security, and care policies, are crucial for ensuring dignified and sustainable aging in that nation. In summary, the establishment of a regional human rights framework constitutes a fundamental advancement. Moreover, its impact will depend on the commitment and capacity to translate these rights into effective, culturally pertinent, and sustainable public policies that mitigate inequalities while promoting autonomy, functional ability, and equitable access to care.

#### 3.3.1. The Organization of American States and the Inter-American Human Rights System in Advancing Healthy Aging

The OAS was founded in 1948 in Bogotá, Colombia [55]. It is composed of thirty-five independent member states in the Americas [56]. The OAS functions as an intergovernmental forum to promote cooperation based on democracy, human rights, security, and development [55]. In the same year, the OAS adopted the American Declaration of the Rights and Duties of Man [57]. This Declaration established the fundamental principles for the protection of human dignity. It also served as a key antecedent of the IAHRS.

In particular, the Inter-American Commission on Human Rights (IACHR) plays a central role. This organism was created in 1959 to promote and defend human rights throughout the Americas [58]. Its main functions include promoting human rights awareness, making recommendations to member states, and processing individual petitions [58,59]. In cases of grave and urgent situations, the IACHR can grant precautionary measures [59]. It can also refer matters to the Inter-American Court of Human Rights (I/A Court HR) [60]. These mechanisms reinforce the international responsibility of States. They enhance the protection of older adults by ensuring that dignity, equity, and inclusive environments remain central to public action. This aligns fully with the principles of healthy aging.

#### 3.3.2. Background on the Protection of Human Rights for Older Persons in the Americas

The protection of older adults’ human rights in the Americas has advanced through legal documents. These instruments predate the adoption of specific aging-related treaties. The historical trajectory shows a clear commitment to dignity and well-being of this population group. Since the mid-twentieth century, the IAHRS has acknowledged the need to protect older adults.

Three foundational instruments established the regional human rights framework. The American Declaration of the Rights and Duties of Man recognized the right to social security for subsistence [57]. The American Convention on Human Rights prohibited the death penalty for persons aged seventy or older [60]. The Protocol of San Salvador expanded protection by establishing social security rights (Article 9). It also obligated States to provide food and medical care (Article 17) and promote labor market inclusion [61]. Additionally, the Inter-American Convention on the Prevention, Punishment, and Eradication of Violence against Women explicitly includes older women as a vulnerable group [62].

Beyond continental instruments, subregional initiatives in Latin America and the Caribbean have contributed to the protection of older people’s rights. Among these, the Regional Strategy for the Implementation in Latin America and the Caribbean of the Madrid International Plan of Action on Aging (2003) promotes dignified and safe aging [63]. Others are the Brasilia Declaration (2008) [64], the Plan of Action of the Pan American Health Organization (2009) [65], and the San José Charter on the Rights of Older Persons in Latin America and the Caribbean (2012) [66].

Several international instruments also contribute to the protection of older people. The United Nations Principles for Older Persons (1991) set forth recommendations intended to ensure independence, participation, care, self-fulfillment, and dignity [67]. The Proclamation on Aging (1992) emphasizes the importance of promoting awareness of the aging population. It recommends that States develop policies and programs directed toward older people. It also fosters collaboration between governmental and non-governmental organizations in health, welfare promotion, and self-help initiatives [68]. For its part, the Political Declaration and Madrid International Plan of Action on Aging (2002) focus on promoting health and well-being in aging within enabling and supportive environments [69]. They encourage governments and non-governmental organizations to reorient social perceptions of older adults, their roles, and the care they require, among other considerations.

In particular, the IACHR played a significant role in the legal analysis by submitting a report to the OAS in 2013. This report served as the basis for the establishment of the Inter-American Convention on Protecting the Human Rights of Older Persons (IAConvPHROP). This instrument was adopted by the OAS General Assembly in 2015 and subsequently entered into force on 11 January 2017 [10]. The convention establishes obligations for the States that either ratify or accede to it.

#### 3.3.3. The Inter-American Convention on the Protection of the Human Rights of Older Adults

To date, thirteen countries have ratified or acceded to the IAConvPHROP, including Argentina, Brazil, Chile, Colombia, Costa Rica, Mexico, Peru, and Uruguay. The Convention, in Article 1, establishes the purpose of promoting, protecting, and ensuring that older adults fully and equally enjoy all their rights and fundamental freedoms. This objective promotes their inclusion, integration, and active participation in society, directly supporting healthy aging and social participation.

States that ratify or accede to the Convention assume substantial obligations. They must establish national mechanisms to ensure the protection of the human rights and fundamental freedoms of older people, as well as enact and enforce specific legislation to prevent abuse, mistreatment, violence, abandonment, and neglect. Furthermore, the IAConvPHROP stipulates that States must develop public policies focused on aging. It is important to note that policies for the protection of older adults must be developed within the framework of IAHRS [10].

In accordance with Article 4 of the IAConvPHROP [10], States Parties commit to safeguarding the human rights and fundamental freedoms of older adults. This commitment is to be carried out through the adoption of legislative, administrative, judicial, and budgetary measures, as well as through the promotion of initiatives aimed at protecting the rights of older adults.

Among the fundamental rights recognized and protected by the IAConvPHROP [10] are the right to equality and non-discrimination based on age (Article 5), as well as the right to life and dignity of this segment of the population (Article 6). Under this Convention, States Parties shall design and adopt policies, actions, and programs aimed at promoting and facilitating the right to independence and autonomy of older people (Article 7). Likewise, participation and integration in community, society, and family life are rights that States are obliged to strengthen (Article 8). One of the most serious phenomena affecting this age group is abuse, which takes various forms, including physical, sexual, psychological, financial, property-related abuse, as well as abandonment. In this regard, the Convention protects the right to security and to a life free of violence (Article 9), with States Parties committing themselves to prevent, investigate, and punish any act of violence against older people. It also prohibits torture and cruel, inhuman, or degrading treatment or punishment (Article 10), ensuring safe environments.

In the field of health, when older people require treatment, intervention, or participation in procedures for research purposes, their free and informed consent must be obtained in a clear, adequate, and transparent manner (Article 11). In addition, the Convention recognizes the right to physical and mental health (Article 19), as well as the right to receive care for protection, health promotion, food security, housing (Article 24), clothing (Article 12), and social security that ensures sufficient income to lead a dignified life (Article 17). It further guarantees the rights to work and education without discrimination (Articles 18 and 20), to cultural and artistic life (Article 21), and to recreation, leisure, and sport (Article 22). Together, these rights contribute to holistic well-being and active aging. The Convention also affirms the recognition of legal personality (Article 30), which enables access to justice (Article 31). It establishes mechanisms of protection to ensure its implementation and monitoring by States Parties (Article 33). These provisions consolidate a comprehensive framework that integrates health policies, health promotion, and healthy aging into protection of older people’s rights in the Americas.

### 3.4. Protection of the Rights of Older Persons in Mexico

**Figure 1:**
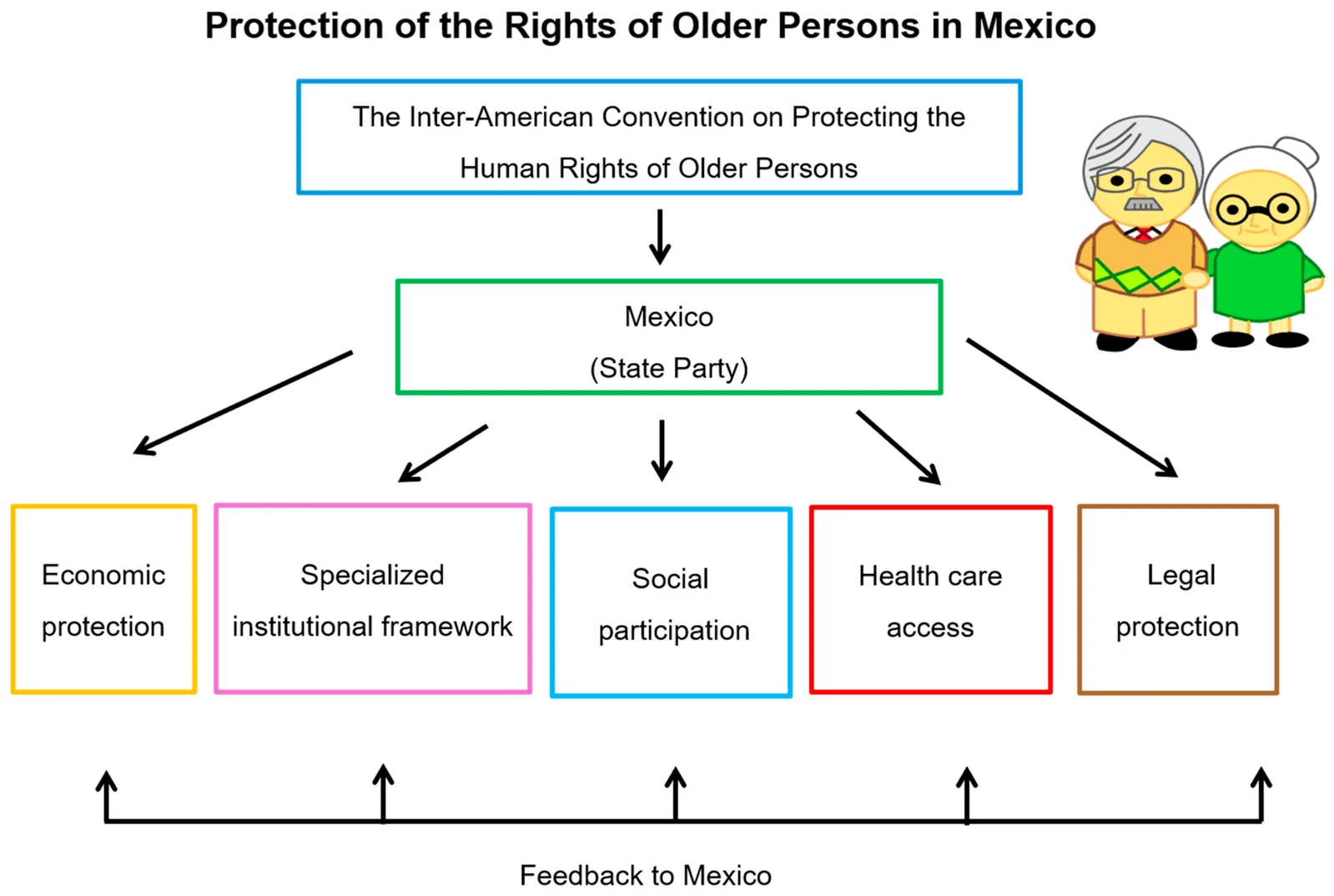
Mexico’s protection of older adults’ rights. The framework comprises five interconnected areas: economic security, specialized institutions, social participation, healthcare access, and legal protection. The structure is aligned with the Inter-American Convention on Protecting the Human Rights of Older Persons. Source: authors’ elaboration.

Mexico deposited its instrument of accession to the IAConvPHROP on 28 March 2023, thereby committing to the protection of this vulnerable population (Figure 1).

#### 3.4.1. Sociodemographic Profile of the Older Population in Mexico

In Mexico, The National Institute of Statistics and Geography (INEGI) has estimated that the total population is approximately 129,477,554, of which around 14% are older persons aged 60 and over [70]. According to the National Survey on Health and Aging in Mexico [71], there are 16, 757, 590 persons aged 60 and over (9,115,288 women and 7,642,302 men). Within this population: (a) approximately 17% have no formal schooling, about 67% have between 1 and 9 years of formal education, and around 14% have completed 10 years or more; (b) with regard to marital status, approximately 62% live with a partner (married or in a common-law union), while 38% do not have a partner (single, widowed, divorced, or separated); (c) in terms of economic activity, 32% are currently employed, while 68% are not working, are engaged in unpaid activities (household chores, job search), or are retired/pensioners; and (d) regarding self-reported health status, approximately 6% consider their health to be excellent or very good, 26% good, 50% fair, and 8% poor.

These demographic indicators reveal the economic and social vulnerability of the older population. This underscores the need for the State to fulfill its IAConvPHROP commitments regarding social security, protection against violence, and access to health services. These data also highlight a significant aging of the population, characteristic of the demographic transition in Mexico and Latin America, with important implications for social policies, health, and economic security. Although Mexico’s population remains relatively young, the trend toward aging is accelerating, putting increasing pressure on social and health systems.

The gender distribution indicates longer life expectancy for women, emphasizing the need for specific policies addressing economic insecurity experienced by older women living alone. In addition, older adults in Mexico generally have low education levels, limiting access to specific services, comprehension of rights, and employment opportunities, as well as active participation in the economy and society. It is suggested that this group is particularly vulnerable to loneliness and economic and social dependence, particularly women, who tend to outlive their partners, as approximately 38% of them do not have a partner.

In terms of economic activity, most older adults depend on pensions, family assistance, or government programs. So, it is important to distinguish the need for social protection and economic security policies for this demographic group. Likewise, approximately one in three older adults continue to work, a phenomenon that can be attributed to both economic necessity and inadequate pension coverage. Most subjects were classified as having fair health. This is a challenge to the access to health services, preventive care, and quality of life. It also underscores the need for health policies tailored to older adults. Thus, the combination of low educational attainment, economic dependence, and fair or poor self-reported health renders this population particularly vulnerable. Therefore, this group requires comprehensive policies on social protection, health promotion, and human rights to ensure safe environments and dignified aging.

However, these conditions vary considerably across regions. While these national averages highlight general patterns, they do not capture substantial territorial heterogeneity. The proportion of older adults varies significantly across states. Mexico City reports the highest concentrations due to low fertility rates. Veracruz shows similar levels, primarily attributable to the out-migration of younger working-age populations [72]. In contrast, southern states such as Chiapas and Oaxaca have lower proportions of older adults. This reflects higher fertility rates and younger population structures [72]. The incidence of poverty among older adults follows a comparable regional pattern. Southern states have the highest poverty rates, e.g., Oaxaca reaches 57.5%, and Chiapas 54.8%. Meanwhile, northern states exhibit the lowest rates, with Baja California Sur reporting 12.8% [72]. This overlap reflects the convergence of younger population structures with the highest old-age poverty in southern states. This suggests that future cohorts will age under even more adverse conditions [73]. These disparities suggest that uniform national policies may be insufficient. This underscores the need for place-based approaches adapted to each region’s specific demands [74]. For instance, rural southern regions could benefit from expanded mobile health units and supplementary cash transfers. Investment in age-friendly infrastructure and long-term care facilities should be prioritized in central and northern urban areas [73,75].

#### 3.4.2. Background of Social Policy for Older Adults in Mexico

Mexico has made notable advances in the formulation of public policies intended to protect its older adult population. Historically, the country’s demographic profile was predominantly young, characterized by high fertility rates and a relatively small proportion of older adults. Within this context, the State began to design targeted strategies for this demographic group. In 1979, the National Institute for the Elderly (INSEN) was established as a federal government agency under the Ministry of Health and Assistance, with the purpose of supporting older people in coordination with the Ministries of Labor, Public Education, and Human Settlements. Its functions included providing employment opportunities and vocational training, disseminating guidance and information regarding shelters and recreation centers, and promoting recreational activities, among other initiatives [76]. At that time, prevailing perceptions characterized older adults as individuals in need of social assistance due to physical, social, and economic vulnerabilities. Consequently, social policy was mainly structured around a welfare-oriented approach, emphasizing poverty alleviation and social integration. Nevertheless, the scope of these efforts remained limited by budgetary capacity.

In 2002, INSEN was transformed into the National Institute for Older Adults (INAPAM) and incorporated into the Ministry of Social Development (SEDESOL). This occurred as the social implications of aging became increasingly evident. INAPAM’s initiatives involved multiple federal agencies, including the Ministries of the Interior, Finance and Public Credit, Education, Labor and Social Welfare, Communications and Transportation, and Tourism, to comprehensively implement public policies for this age group. This institutional change marked a paradigm in which older individuals were regarded as rights-bearing individuals [76,77,78,79]. It represented progression from a welfare assistance framework to one that integrates health policy, social protection, and active aging.

This approach was consolidated with the enactment of the Law on the Rights of Older Persons in 2002, which sought to regulate public policies by formally integrating the rights of older adults [80]. In line with this, the law also includes the capacity to demand the guarantee of their welfare, social participation, autonomy, and access to services and benefits on equal conditions. This law reinforced the integration of health policies and social programs aimed at promoting healthy, safe, and active aging in Mexico.

#### 3.4.3. Social Policies and Programs for Older Population in Mexico

In Mexico, social policies and programs for older adults promote comprehensive well-being, economic security, and access to essential services. Notably, the Pension for the Welfare of Older Persons provides financial support to individuals aged 65 and over. It is a universal program administered by the Government of Mexico [81]. This pension reduces food insecurity by 7.3% in older adults living alone, and by 10.1% for those lacking social assistance [82]. These findings indicate that economic transfers can reduce material deprivation in isolated older populations. However, this evidence is mixed to specific outcomes since it does not address health, functional ability, or quality of life. Some studies have found significant variations in the program’s effects across rural and urban areas, as well as by gender [74]. These findings show the importance of evaluations that consider geographic and demographic factors.

In the health sector, IMSS-Bienestar provides free medical services to individuals without social security coverage. INAPAM promotes active aging and the defense of older adults’ rights. It also grants benefits such as discounts on services and cultural activities [83]. Likewise, the National System for the Integral Development of the Family (SNDIF) offers comprehensive care through gerontological centers. These places provide medical, psychological, and assistance services [84,85]. Furthermore, affordable housing programs such as Well-Being Housing prioritize access to decent housing for vulnerable older adults [86].

Nonetheless, ensuring care continuity for older adults through IMSS-Bienestar will require strengthened intergovernmental coordination and sustained fiscal planning [87]. INAPAM’s institutional reach operates within the context of available resources and a family-based care model [88]. Similarly, SNDIF gerontological centers have experienced variability in coverage, suggesting opportunities for strengthening capacity.

In addition to these structural programs, the Health Door-to-Door program offers home-based medical care to older adults and individuals with disabilities. This initiative improves access for vulnerable communities [89]. However, its implementation requires robust information systems to monitor service delivery and ensure continuity of care. In Mexico, ongoing efforts have focused on developing strategic information systems to support aging-related policies [90].

In the legal sphere, free legal advice is available in inheritance, alimony, and domestic violence matters. At the same time, recent legislation seeks to prevent fraud and property dispossession. The National Human Rights Commission (CNDH), together with state commissions, guarantees human rights protection. It also promotes initiatives aimed at fostering social inclusion, recreation, and economic participation among older individuals. These legal measures support public health strategies by guaranteeing safe conditions and equal access to rights, thereby advancing the goals of healthy aging.

The viability of these initiatives depends on a solid fiscal foundation. The fiscal burden of non-contributory pensions has increased significantly, necessitating proactive fiscal planning to ensure long-term sustainability [91].

#### 3.4.4. Legal and Policy Framework for the Protection of Older Adults in Mexico

Mexico is committed to safeguarding the rights of older adult persons, defined as individuals aged 60 and above. This commitment is reflected in the various international treaties that the country has signed or ratified, which establish the obligation of the Mexican State to guarantee the rights of older adults. Among these instruments are the Universal Declaration of Human Rights [92], the International Covenant on Economic, Social and Cultural Rights [93], the International Covenant on Civil and Political Rights [94], R162-Older Workers Recommendation, 1980 (No. 162) [95], the Declaration on the Elimination of Violence against Women [96], the American Convention on Human Rights [60], Protocol of San Salvador [61], IACPHROP [10], among others. Together, these international instruments constitute a comprehensive legal framework that guides the Mexican State in promoting health equity, protecting rights, and guaranteeing conditions for healthy aging.

Mexico has made progress in protecting the population aged 60 and over by guaranteeing their social, human, and legal rights through various federal-level legal instruments. It is important to note that Mexico is a federated state, meaning each federative entity has its own legal system, which must be aligned with federal legislation as well as with treaties, conventions, and international organizations to which Mexico is a part. This multi-level alignment ensures consistency in health policies and social protection measures that directly impact older adults’ well-being. The following outlines the federal-level legal framework in Mexico for the protection of older population:

(i) The Political Constitution of the United Mexican States: establishes the right to non-discrimination based on age, as well as the right to social security and to a non-contributory pension provided by the State [97]. These rights reinforce social protection and economic security, essential for dignified and healthy aging.
(ii) The Law on the Rights of Older Persons: condemns all forms of violence, guarantees health and food security, provides social assistance in cases of unemployment, and recognizes the right to education. It also establishes the family’s responsibility for maintaining and preserving the quality of life of older people [80]. This law integrates health promotion and social participation as pillars of active aging.
(iii) General Health Law: guarantees the right to geriatric health, including mental health [98]. This directly supports preventive care and health promotion strategies for older adults.
(iv) Social Assistance Law: establishes that the older people experiencing socioeconomic deprivation must receive support from the State [99]. This law improves equity in access to services and protection against vulnerability.
(v) Federal Law to Prevent and Eliminate Discrimination: as a discriminatory act the denial of financial services to an older person [100]. By addressing age-based discrimination, this law ensures fair access to resources that sustain healthy aging.
(vi) Federal Criminal Code: protects older adults against discrimination based on sexual orientation or gender identity and increases penalties in cases of femicide, domestic violence, and unlawful deprivation of liberty [101]. These protective measures contribute to safe environments, which are essential for healthy and dignified aging.
(vii) National Code of Civil and Family Procedures: in family and civil matters, older people are afforded protection without altering the procedural order [102]. This legal provision ensures procedural fairness and access to justice, which is a key factor in equity and healthy aging.
(viii) Federal Civil Code: allows older persons to be excused from exercising parental authority, guardianship, executorship, and other similarities among roles [103]. By recognizing functional limitations and protecting autonomy, this measure aligns with health policies that promote dignity and safe aging.

In Mexico, a federal legislative initiative aims to safeguard the right to dignified and equitable caregiving, ensuring the provision of quality services for older adults, children, and individuals with disabilities or chronic illnesses. The proposal underscores the principle of shared responsibility among the State, families, and society, framing caregiving as a fundamental component of health promotion, social justice, and the creation of supportive environments for healthy aging.

Despite this comprehensive legal framework, significant gaps persist between the law and its implementation. Institutional barriers that limit access to services and protections for older adults have been documented [104]. It is recommended to strengthen enforcement mechanisms, and addressing rights violations will depend on enhanced institutional capacity and training of judicial personnel [105]. Mexico has not consolidated a comprehensive national long-term care system, unlike Uruguay, Argentina, Costa Rica, and Chile, which have established care strategies for older adults [106,107]. This absence perpetuates inequalities and places disproportionate care burdens on families, especially on women, who perform 75.1% of caregiving tasks [108].

The long-term care infrastructure remains incipient. Although the Mexican Social Security Institute inaugurated its first Day Center for older adults with functional dependency in 2022, this represents a pilot initiative rather than a systemic response [108]. Gerontological centers and day stays serve a broad population but lack specialization for complex care needs [109]. Private nursing homes operate with limited oversight, and 98% of personnel have no formal training [108]. Residential care homes exhibit challenges including limited health personnel, few recreational activities, and gaps in basic needs support [110]. Studies in day centers have identified the need for strengthened family support mechanisms, as some cases of mistreatment have been documented in family environments [105].

These systemic gaps have measurable consequences. Some studies show that older adults with functional limitations are up to 23% more likely to be hospitalized and tend to have longer stays [111]. The Inter-American Development Bank estimates that providing adequate long-term care could reduce unnecessary hospitalization days, generating significant cost savings for the health system [112]. Institutionalized older adults report high levels of familial loneliness, social isolation, and feelings of abandonment and depression [110].

Effective reform requires integrating health and social care systems. This includes developing regulations for a national long-term care system and for residential facilities. It also requires establishing training and certification programs for care personnel and incorporating community-based approaches [107]. Success will depend on adequate resource allocation, systematic training of judicial and administrative personnel, and robust monitoring mechanisms capable of tracking compliance.

## 4. Discussion

This discussion is organized around three domains: legal protection, social determinants, and public health policies (Figure 1). Together, these domains shape the outcomes of healthy aging in Mexico.

Mexico’s accession to the IACPHROP represents a significant step forward in the protection of older adults’ rights. This instrument establishes obligations for the Mexican government to promote, protect, and guarantee the rights [10]. Consequently, Mexico is strategically positioned to consolidate a comprehensive approach to population aging. Nevertheless, Mexico’s Law on the Rights of Older Persons was enacted in 2002. In this regard, periodic normative reviews would facilitate harmonization with evolving international standards [113]. Furthermore, the translation of regulatory provisions into effective practice requires continued attention. This implementation gap is not unique to Mexico, as it is commonly observed in Latin American jurisdictions [114]. Therefore, strengthening monitoring is essential to ensure legal advances to improve older people’s well-being.

Beyond legal frameworks, Mexico’s policy trajectory must be analyzed through the WHO Healthy Aging Framework, which shows a pattern of asymmetric progress. The framework defines healthy aging as a continuous process of developing and preserving functional ability. This results from the interaction between personal resources and factors that support or constrain daily life [21]. An examination of universal pensions, healthcare expansion, long-term care services, and regional disparities highlights a central tension. Mexico has advanced considerably in reinforcing the personal determinants of functional capacity, particularly economic stability and access to medical services. In contrast, progress in improving contextual determinants remains an area requiring further development. These include support networks, institutional coordination, and the accessibility of physical environments. As described below, this imbalance generates a gap in effective capacity. Such conditions limit the conversion of legal rights and economic resources into outcomes of dignified, autonomous, and healthy aging. This asymmetry is relevant in the context of the WHO Decade of Healthy Aging (2021–2030). The initiative promotes coordinated action on ageism, age-friendly environments, and long-term care. In this regard, Mexico continues strengthening efforts across these domains, though ongoing structural challenges require sustained institutional commitment.

### 4.1. Economic Protection

The universal non-contributory pension constitutes a notable achievement in Mexican social policy. It improves economic security for millions of older adults [81]. Nevertheless, its long-term sustainability deserves careful examination, as public universal pensions have increased in recent years [91]. Accordingly, demographic projections indicate that proactive fiscal planning will be essential to maintain the program’s benefits while balancing other social priorities [91]. Additionally, Mexico’s high labor informality rate constrains the contributory base for social security. This places greater pressure on non-contributory schemes, which are more vulnerable to reform and fiscal volatility [115,116]. Therefore, sustainable financing mechanisms, including potential fiscal reforms or gradual benefit adjustments, could help ensure long-term viability. Mexico’s universal model is notable within Latin America. It is comparable to Chile’s quasi-universal scheme [81,117,118,119,120,121]. However, this achievement may face viability risks if high levels of labor market informality persist. This is because precarious employment remains a structural challenge across the region. Moreover, the long-term stability of Mexico’s defined-contribution pension system is equally concerning. The financial equilibrium of individual accounts is undermined by low contribution densities and insufficient accumulation periods [49]. Such structural weaknesses could compromise the system’s capacity to provide adequate retirement incomes. This scenario would necessitate difficult policy decisions, including adjustments to contribution rates, retirement ages, or benefit formulas.

The comparative data from Table 2 reveals a significant paradox. Mexico’s universal pension scheme attains coverage comparable to Chile’s (>75%). However, this is accompanied by lower health expenditure (6% vs. 11% of GDP). Furthermore, the out-of-pocket burden is substantially higher (41% vs. 15% in Colombia). These discrepancies raise fundamental questions about equitable medical access. Specifically, they question whether pension-derived income effectively facilitates care. This concern is particularly salient, given the high prevalence of chronic conditions in this age group [122]. The heavy dependence on household spending suggests pensions may be insufficient to cover essential medical needs. Consequently, this economic fragility risks undermining the program’s health-promotion objectives. These economic challenges are compounded by specific health policy gaps.

### 4.2. Health Policies

Evidence on specific health policies to optimize effectiveness remains mixed and varies across interventions. ENSANUT Continua 2023–2024 provides recent data. It reveals that 39.7% of the older population is living with multimorbidity, while 34.5% report depressive symptoms [122]. Additionally, 14.8% of older adults present frailty, and 27.4% experience functional dependence in daily living [122]. These statistics show the urgent need for tailored health policies that address the complex needs of an aging population.

These health challenges are compounded by significant social and economic vulnerabilities, as reported by Mexico’s National Council for Social Development Evaluation (CONEVAL). According to CONEVAL’s 2024 report, 3.9 million of this age group live in poverty (31%), and 29.4% lack access to health services. Food deprivation has also been reported in households headed by older people with 15.5% experiencing food insecurity. This proportion rises to 23.7% in rural areas [123].

In response, Mexico has implemented several social programs, most notably the universal non-contributory pension, which has shown measurable health benefits. It reduces food insecurity by 7.3% for isolated older adults and 10.1% for those without social support [82]. It improves nutrition and mental health, particularly in women, indigenous populations, and the poorest beneficiaries [122]. It also reduces rural female mortality by 5.5% [124]. Nevertheless, the effect is not uniformly positive, as it depends on payment frequency. A monthly state pension reduced frailty in women, while the bimonthly federal pension was associated with increased frailty in women [125]. This suggests that payment timing affects how resources are spent on regular food purchases versus durable goods. Importantly, the impact of the pension on functional ability has been partially evaluated [125], but its effects on quality of life still lack rigorous, systematic assessment. This evidence gap is particularly concerning given the high level of economic dependence on social programs. For example, 39.9% of older adults rely on these programs as primary income (50.6% women and 27.3% men) [72].

To address these limitations, Mexico could take lessons from other Latin American countries to implement effective aging policies. Costa Rica, Colombia, and Brazil offer primary and community care strategies that could guide Mexico’s approach [121]. In this regard, Mexico has not consolidated a comprehensive national long-term care system, a critical gap that perpetuates family reliance without adequate institutional support. Uruguay and Costa Rica have established care strategies that integrate health and social services, offering valuable models [126].

These examples indicate that Mexico must address healthcare workforce gaps through expanded professional training. Priority areas include geriatrics, gerontological nursing, and community-based care, which remain insufficient for an aging population. The region’s cultural tradition of intergenerational solidarity can complement the formal care infrastructure [127]. Nevertheless, policies that promote community participation require adequate resources to ensure that caregiving responsibilities do not place a disproportionate burden on families or women [128]. In addition to direct health services, institutional frameworks also shape aging outcomes.

### 4.3. Institutional Frameworks and Social Participation

Specialized institutions constitute another shared focus across the region. Mexico, Uruguay, Argentina, Colombia, and Costa Rica have established agencies dedicated to the well-being of older people. These entities coordinate programs and promote community integration [121]. Social participation and active aging are central priorities in Mexico, Uruguay, Colombia, Brazil, Chile, and Cuba. Programs aim to keep older adults integrated into community, cultural, and family life while promoting autonomy and intergenerational interaction [129]. Several nations have adopted legal protections to ensure equality and prevent abuse [114,121]. In Mexico, the Law on the Rights of Older Persons (2002) together with community support networks demonstrate these principles [80]. Barbados and Trinidad and Tobago illustrate how regulatory frameworks can be adapted to diverse cultural contexts [114]. The cultural narratives surrounding aging in the region deserve thoughtful consideration. Intergenerational solidarity is widely recognized as a cultural strength that supports family-based care models. This tradition can complement, rather than substitute, formal care infrastructure. Policies that promote community participation are valuable. However, they should be accompanied by adequate resources. This ensures that caregiving responsibilities are distributed equitably. Furthermore, it prevents a disproportionate burden on families, particularly women.

### 4.4. Regional Heterogeneity

Regional heterogeneity within Mexico is an important consideration at the policy level. Health and economic conditions vary considerably from one state to another. In 2024, 46.5% of urban older people had health coverage, compared to 16.3% in rural areas [130]. The southern states have the highest poverty rates and the worst health indicators [130]. Uniform national programs may not effectively address local needs. Territory-based approaches that respond to territorial diversity could improve policy responsiveness. The urban–rural coverage contrast suggests national programs need local adaptation [130]. Decentralization should be accompanied by capacity at subnational levels. A phased approach that strengthens institutional capacity could help reduce, rather than exacerbate regional disparities. Public policies require territorial and culturally relevant approaches to address diversity, while maintaining legal safeguards and social equity. Therefore, it is important to consider emerging digital health technologies, including telehealth and remote monitoring. These innovations may reduce access gaps in rural areas, but implementation requires infrastructure and digital literacy investments for this age group.

### 4.5. Toward an Integrated Policy Framework

The progress made by Mexico and the region can be analyzed through five key areas: economic protection, specialized institutions, social participation, access to health care, and legal protection [114]. Some authors have emphasized that coordination across institutional reports and mechanisms is essential for effective policy implementation [107]. Mexico’s aging transition is occurring at a relatively lower level of economic development. This is compared to regions that aged earlier, creating unique challenges [121]. While Mexico can learn from countries with more established policies, its specific socioeconomic context requires tailored solutions. Regional cooperation and exchange of best practices enrich national strategies [2,113]. Aging is understood as an opportunity to rethink sustainable development and social cohesion. Mexico and the region are well-positioned to promote a model of social justice and intergenerational solidarity. This contributes to more inclusive and resilient societies.

### 4.6. Policy Implications and Cross-Country Lessons

Mexico’s trajectory offers lessons applicable to other middle-income nations. These countries face rapid demographic aging with structural informality and fiscal capacity [49]. Mexico’s experience shows that universal non-contributory pensions can achieve broad coverage rapidly. However, long-term viability depends on parallel labor market formalization. This challenge is prevalent in Latin America, where informal employment for older population is high in most countries [49]. A critical aspect is that the long-term care landscape in Mexico is fragmented, which exposes a fundamental gap. This means that legal rights and financial resources are inadequate without investments in care infrastructure and workforce professionalization. Three strategic priorities emerge for policymakers in comparable circumstances. First, fiscal planning must incorporate aging-related considerations without displacing other social investments. Second, develop a locally adapted care model to leverage community and family networks while providing institutional support and caregiver training. Third, invest consistently in longitudinal data systems to facilitate monitoring of policy effectiveness across populations. These priorities must be contextualized in accordance with the institutional capacity, cultural norms, and demographic trajectory of each country. Furthermore, the fiscal implications of aging population extend beyond economic consequences. These include the macroeconomic costs of unpaid care work, changes in productivity, and a contraction of the labor force. These factors need comprehensive policy responses that encompass employment, health, and social protection. Ultimately, Mexico’s case underscores an important lesson. For policies on aging to work, they need more than legal frameworks and social programs. Political commitment is required for the demographic transition, along with cross-sectoral coordination and adaptive governance.

### 4.7. Limitations of the Study

This narrative review is based on documentary analysis, not primary data. It does not include detailed evaluation of the policies under consideration. As a narrative review, it is subject to inherent methodological limitations. Potential publication bias arises because positive findings are more often published. Selection bias may result from predefined search strategies and database choices. Relevant evidence may be omitted, including unpublished evaluations or grey literature. Recent policy changes outside the search timeframe may also remain unexamined. No formal and systematic assessment of the quality of the included sources was conducted.

Moreover, it does not examine older indigenous persons or rural residents in depth. This limits understanding of cultural and territorial differences. Future research must integrate these dimensions for broader analysis. This would provide a more enriching view of diverse contexts.

The regional comparison is based on bibliographic sources and may simplify diverse national experiences. The analysis focuses on the federal level without examining state differences. Future research should use case studies and more in-depth comparative analyses. Mixed methods integrating quantitative and qualitative evidence are also required. This would facilitate observation of gradual program implementation over time. It would also enable precise evaluation and adjustment of public policies. External factors may also adversely affect the implementation of these policies. For example, earthquakes, hurricanes, or pandemics can disrupt essential services. Such events challenge even well-intentioned governmental efforts to protect older people.

## 5. Conclusions

Mexico has advanced in protecting older adults’ rights through legislation and policy. Moreover, its accession to the IACPHROP confirms Mexico’s commitment to international standards. Thus, Mexico is situated within a framework of legal and political responsibility. Furthermore, non-contributory pensions, health programs, and social inclusion initiatives have positively influenced several aspects of older adults’ well-being. However, the evidence on their overall effectiveness is heterogeneous. These initiatives will depend on fiscal capacity over the coming decades, given Mexico’s demographic transition. Nonetheless, regional experience provides lessons, while domestic instruments remain fundamental. Consequently, Mexico can reinforce these instruments through cooperation, systematic evaluation, and targeted adjustments. At the same time, placing Mexico in the Latin American context offers perspectives. Importantly, aging should be seen as an opportunity, not solely a challenge of protection. Older people also contribute knowledge, experience, health promotion, and community cohesion. Therefore, integrating their perspectives into public policy is essential for inclusive societies.

To translate these commitments into tangible outcomes, the following actions are recommended: (i) policymakers should implement fiscal reforms to ensure pension financial resilience. They should also adopt place-based approaches to reduce regional disparities; (ii) healthcare providers must strengthen geriatric training and expand community-based primary care; (iii) researchers should conduct mixed-methods evaluations of policy impacts on health outcomes, including functional ability; (iv) future legislation must harmonize national laws with IACPHROP standards and regulate care services; (v) long-term care planning requires a comprehensive system integrating health and social services under quality standards; and (vi) public health policy should strengthen monitoring mechanisms and adopt intersectoral approaches addressing social determinants of health.

## Figures and Tables

**Table 1:** Non-contributory pensions for older adults in Latin America and the Caribbean ^1^.

Country	Universal or Targeted	Mandates the Absence of Contributory Pensions
Bolivia (Plurinational State of)	Universal	No
Guyana	Universal	No
Mexico	Universal	No
Chile	Quasi-Universal *	No
Antigua and Barbuda	Targeted	Yes
Argentina	Targeted	Yes
Bahamas	Targeted	Yes
Belize	Targeted	Yes
Brazil	Targeted	Yes
Colombia	Targeted	No
Costa Rica	Targeted	Yes
Cuba	Targeted	Yes
Dominican Republic	Targeted	Yes
Ecuador	Targeted	No information
El Salvador	Targeted	Yes
Guatemala	Targeted	Yes
Panama	Targeted	Yes
Paraguay	Targeted	Yes
Peru	Targeted	Yes
Saint Kitts and Nevis	Targeted	Yes
Saint Vincent and the Grenadines	Targeted	No
Trinidad and Tobago	Targeted	Yes
Uruguay	Targeted	Yes
Venezuela (Bolivarian Republic of)	Targeted	No

^1^ Source: Prepared by authors based on data from the Economic Commission for Latin America and the Caribbean (reference [36]). * Covers all older adults except the wealthiest 10%.

## Data Availability

No new data were created or analyzed in this study. Data sharing is not applicable to this article.

## Abbreviations

The following abbreviations are used in this manuscript:

WHO	World Health Organization
IAHRS	Inter-American Human Rights System IAHRS
IACPHROP	Inter-American Convention on Protecting the Human Rights of Older Persons
OAS	Organization of American States
ECLAC	Economic Commission for Latin America and the Caribbean
IACHR	Inter-American Commission on Human Rights
I/A Court HR	Inter-American Court of Human Rights
IAConvPHROP	Inter-American Convention on Protecting the Human Rights of Older Persons
INEGI	National Institute of Statistics and Geography
INSEN	National Institute for the Elderly
INAPAM	National Institute for Older Adults
SEDESOL	Ministry of Social Development
SNDIF	National System for the Integral Development of the Family
CNDH	National Human Rights Commission
IBRD	International Bank for Reconstruction and Development
OECD	Organization for Economic Co-operation and Development
ILOSTAT	International Labour Organization Statistics
WCDGHC	Wittgenstein Centre for Demography and Global Human Capital
GDP	Gross Domestic Product
CHE	Current health expenditure
UHC	Universal Health Coverage
